# Reproductive success of Eastern Bluebirds (*Sialia sialis*) varies with the timing and severity of drought

**DOI:** 10.1371/journal.pone.0214266

**Published:** 2019-08-09

**Authors:** Reneé E. Carleton, John H. Graham, Adel Lee, Zachary P. Taylor, Jon F. Carleton

**Affiliations:** 1 Department of Biology, Berry College, Mount Berry, Georgia, United States of America; 2 Etosha Business and Research Consulting, LLC, Rome, Georgia, United States of America; 3 Department of Environmental Science, Berry College, Mount Berry, Georgia, United States of America; 4 MIService Consulting, Taylorsville, Georgia, United States of America; University of Tulsa, UNITED STATES

## Abstract

Drought affects avian communities in complex ways. We used our own and citizen science-generated reproductive data acquired through The Cornell Lab of Ornithology’s NestWatch Program, combined with drought and vegetation indices obtained from governmental agencies, to determine drought effects on Eastern Bluebird (*Sialia sialis* L.) reproduction across their North American breeding range for the years 2006–2013. Our results demonstrate that some aspects of bluebird reproductive success vary with the timing and severity of drought. Clutch size was unaffected by any level of drought at the time of clutch initiation or during the 30 to 60 days prior to clutch initiation. Hatching and fledging rates decreased as drought severity increased. Drought conditions occurring at least 30 days prior to the date eggs should have hatched and 60 days prior to the date offspring should have fledged negatively affected reproduction. We also demonstrate the value of datasets generated by citizen scientists in combination with climate data for examining biotic responses at large temporal and spatial scales.

## Introduction

Drought in varying degrees of severity and duration has affected the North American landscape and its biota for many hundreds of years [1, 2]. The lack of available soil moisture typical of drought can reduce or eliminate vegetation serving as either food or habitat for birds and their prey [3–5]. As a result, drought can negatively influence breeding birds dependent on those resources for their survival and reproduction. Unsurprisingly, a reduction in breeding success is one of many consequences [3, 6–8]. Dispersal away from drought-stricken areas [9], increased mortality of adults, their offspring, or both adults and offspring, nest abandonment [3, 10], and reduced breeding attempts [3, 7, 10], have all been reported as a direct result of drought. Albright et al. [8], for example, found drought to have diverse effects on bird-species abundance, with long-distance migratory species and those residing in semi-arid areas more severely affected than montane species, short-distance migrants, or synanthrophes.

Prediction models for the 21st century indicate drier conditions and periods of persistent drought for North America [11, 12]. Models also suggest that more than 50% of North American bird species will lose half of their distributional range, partly because of climate-associated impacts [13], such as drought. Moreover, because we know that drought negatively impacts many bird populations [7, 14, 15], it is critical to understand how and when drought will impact future reproduction across the entire breeding range of a species.

Detecting changes in species abundance at large spatial and temporal scales, such as the breeding range of a species across multiple years, requires intensive data collection. Few researchers, however, have the time or resources to accomplish such an undertaking. Citizen science, the collection of data by a network of volunteers, is increasingly used as a means of acquiring large data sets over wide geographic areas and long time periods [16–19]. Although some scrutiny of citizen-science data is advisable, proper guidelines can provide for the generation of reliable data [20]. For example, the revelation of a climate-related change in the egg-laying dates of Tree Swallows (*Tachycineta bicolor*) was made possible by use of citizen-science data [21]. One highly successful citizen-science project is The Cornell Lab of Ornithology’s NestWatch Program, officially launched in 2008; it had evolved from the Cornell Nest Record Card program begun in 1965 [22]. NestWatch volunteers record breeding variables for 600 North American breeding bird species. These variables include number of nesting attempts, eggs produced, and young hatched and fledged, as well as nest-location information obtained using online mapping applications [22]. Since the inception of NestWatch, more than 60 peer-reviewed articles using data generated by citizen observers have been published [23]. Large datasets generated by citizen-scientists, in conjunction with standardized climate indicators, are therefore ideally suited to examine climate impacts across the range of a species.

The aim of this study was to use citizen science-generated data from NestWatch, and our own data, in conjunction with the normalized difference vegetation index (NDVI) and North American Drought Monitor (NADM) PDSI-based drought levels, to determine how and when droughts of varying severity affect reproduction across the Eastern Bluebird (*Sialia sialis* L.) breeding range. As secondary cavity-nesting birds, Eastern Bluebirds readily adopt nest boxes provided them by individuals who enjoy hosting birds [24, 25] and, in some areas, nest boxes have replaced scarce natural nesting resources [25]. Inclusion of Eastern Bluebirds as a species monitored under NestWatch provided us with a significant data source for this study.

Geographical variations of clutch size are typical of passerines [20, 26, 27] and especially multi-brooded species [28, 29]. We hypothesized that reproduction of Eastern Bluebirds should follow this pattern, but drought during critical pre-breeding and breeding periods would negatively impact their reproductive success. Our results indicated that drought conditions, regardless of severity, and occurring either pre-breeding or during the clutch formation period, had no significant effect on clutch size, but hatching and fledging rates decreased as severity of drought increased. We also found that the timing of drought conditions also influenced hatching and fledging success.

## Methods

### Reproductive data

We obtained NestWatch (https://nestwatch.org/explore-data/) observation records for Eastern Bluebirds across their breeding range for the years 2006 through 2013. Records included a unique nesting attempt identification number, observer identification number, U.S. state or Canadian province location, latitude and longitude of the nest under observation, year of observation, date of observation, clutch initiation date, clutch size, number hatched, number of young fledged, fledge date, and whether the nesting attempt was successful or unsuccessful. Following importation of all records into a Sequel Query Language (SQL) database, we ran queries to identify and eliminate records of nesting attempts and associated activity outside of the typical March through August breeding season, records where more than 7 eggs were observed in a single nest that might indicate use by more than one female or reuse of a nest containing an abandoned partial clutch, and records where the number of eggs was recorded as 0 but numbers of young, or young fledged was recorded as greater than 0. We also eliminated records for which the recorded fledge date was greater than 40 days past the clutch initiation date; given an expected sequential oviposition, an average 12 to 15 days of incubation, and 20 days between hatch to fledge [30], we judged the reliability of these observations as questionable. Records from states on the breeding range boundaries with fewer than 5 submissions were also deleted as well as those with geographic locations outside the range of Eastern Bluebirds or otherwise questionable. For analyses, we used records with expected hatch dates between Julian days 60 and 243 and expected fledge dates to day 273. We included data of the same NestWatch reproductive variables and time period from our study site located on our home institution’s land tract in Floyd County, GA, where we have monitored Eastern Bluebird reproduction since 2002.

### Ethics statement

Our data collections were carried out in strict accordance with the Ornithological Council’s recommendations [31] and under the permits issued by the U.S. Geological Survey’s Bird Banding Laboratory and the State of Georgia’s Department of Natural Resources. The protocol was approved by the Institutional Animal Care and Use Committee of Berry College (Protocol Number: 2006-13-005).

### Drought indices

#### NADM drought categories

We used latitude and longitude recorded for each nest box to identify the nearest weather station within 40 km and then downloaded NADM archival drought status categories for Julian dates of nesting events during the years 2006–2013 (http://drought.gov). NADM categories are determined using several key indices, including a Vegetation Health Index, Crop Moisture Index, modified Palmer Drought Severity Index, and others [32]. For our analyses, drought status categories were treated as ordinal variables (Table 1).

**Table 1:** Drought categories, palmer drought severity indices (PDSI), descriptions, and possible impacts. Data source: National Drought Mitigation Center. 2016. Accessed November 2018.http://droughtmonitor.unl.edu/MapsAndData.aspx.

Category	PDSI	Description	Possible Impacts
N	–	No drought	
D0	-1.0 to -1.9	Abnormally dry	Going into drought: short term dryness slowing planting or growth of crops or pasture. Coming out of drought: some lingering water deficits; pastures or crops not fully recovered
D1	-2.0 to -2.9	Moderate drought	Some damage to crops or pastures; streams, reservoirs, or wells low
D2	-3.0 to -3.9	Severe drought	Crop or pasture losses likely; water shortages common; water restrictions imposed.
D3	-4.0 to -4.9	Extreme drought	Major crop/pasture losses; widespread water shortages or restrictions
D4	-5.0 and less	Exceptional drought	Exceptional and widespread crop/pasture losses; shortages of water in reservoirs, streams, and wells, creating water emergencies

### Normalized difference vegetation index

NDVI (normalized difference vegetation index) is correlated with net primary productivity (NPP) [33]. We calculated a NDVI value for each nesting site for 30-day periods during the breeding season using 14-day Advanced Very High-Resolution Radiometer (AVHRR) 1-km composites downloaded from the U.S. Geological Survey. We averaged two 14-day composites and determined the NDVI for each nesting site using ArcMap 10.3 (ESRI, Redlands, CA). Standardized NDVI was calculated, following Albright et al. [33], as ${\text{NDVI}}_{\text{std}}=\left({\text{NDVI}}_{\text{current}}-{\text{NDVI}}_{\text{historicalaverage}}\right)÷\sigma_{\text{historical}}$. NDVI_current_ and NDVI_historical average_ are for specific locations. NDVI_std_ makes it possible to compare widely different sites, such as relatively arid and relatively humid locales.

### Statistical analyses

Our primary focus was to determine to what degree Eastern Bluebird reproductive success is impacted by drought. We conducted 3 separate analyses (generalized linear mixed-effect models) in R’s lme4 package, with clutch size, hatching success rate (number of eggs hatched divided by clutch size), and fledging success rate (number of young fledged divided by clutch size) as respective response variables. First, we specified a baseline null model (Model 0), which included the Julian date of clutch initiation, hatching, or fledging, nesting site latitude, longitude, and NDVI_std_. We standardized these numerical explanatory variables to have a mean of zero and standard deviation of one. Since reproductive attempts and success are known to vary within a season and across geographic areas [20, 28, 29], we also included interactions between Julian date and nesting site latitude and Julian date and nesting site longitude.

To study the effect of drought on our respective outcome variables, we considered both current drought status and drought statuses shortly prior to the reproductive event of interest (hereafter referred to as “prior drought”). Current drought status was the drought status at the Julian day of clutch initiation, hatching, or fledging of young, respectively. Pinkowski [24] noted the greatest influxes of bluebirds into their breeding territories occurred during an approximately 30-day period, which was followed by a peak in clutch initiations about 30 days later. Along these lines, we elected to evaluate drought conditions no more than 60 days before clutch initiation as prevailing conditions earlier than this would occur during winter when some populations had migrated to other regions or when breeding territories had yet to be established. We thus considered 30 and 60 days prior to laying, hatching, and fledging as possibly critical time periods for not only nest building, but also when insect populations should undergo increases that would in turn be important for the feeding and survival of nestlings [34, 35].

We studied the effects of drought timing by adding each of the three drought-timing variables to the baseline model separately. For Model 1, we added current drought status (D0 through D4, with N as the reference category) to the baseline explanatory variables. In the next model (Model 2), we substituted current drought status with drought status at 30 days prior to each nesting event and, for the final model (Model 3) replaced the 30-day prior drought status with the drought status 60 days prior to the nesting event. We purposefully elected to study the effects drought timing separately, rather than sequentially, in our models due to a high and significant level of temporal autocorrelation in drought status measures at the nesting sites. We estimated temporal autocorrelation among drought measures by using the continuous autocorrelation function corCAR1 in the call to the R function lme (package nlme) and assigning integer values ranging from 0 to 5 for drought levels N to D4, respectively. In the call to corCAR1, we used “form = ~ Time | AttemptID”, where Time is the time covariate, indicating the times at which drought was measured, and AttemptID is the grouping factor, indicating the unique nesting attempts.

To assess the effect on drought status on each of the three outcome variables, we performed likelihood ratio (LR) tests comparing each of Models 1, 2, and 3 to the baseline model, respectively. The LR tests assessed whether the effect of the drought status at the time of, 30 days prior to, and 60 days prior to the date of a nesting event, respectively, was significantly different from zero (H_0_: *β* = 0). If the LR test results indicated that a drought status variable had a significant effect, then we examined how the different levels of drought affected the reproductive outcomes. For this study, the focus was not to predict the outcome variable or to select the best subset of explanatory variables, but to instead test hypotheses about drought-status variables, and to gain insights into the effect of different levels of drought severity and timing of drought.

We used a generalized linear mixed-effects model (glmer function in the lme4 package for R) and Nelder-Mead estimates of the model parameters. For the analysis with clutch size as the outcome variable, we assumed a Poisson probability distribution, with a log, Xβ = ln(*μ*), link function. In contrast to clutch size, the assumptions of a Poisson distribution were violated for both the number of young hatched and the number of young fledged, because of the large percentage of zero observations. Rather than using the number (or count) of eggs hatched (or young fledged) as the outcome variable, we used the success rate (number of eggs hatched and number of young fledged divided by clutch size). With hatching and fledging rate as the outcome variable, we fitted a generalized linear mixed-model, assuming that the hatching and fledging rates were generated from a Binomial probability distribution, with the logit, Xβ=ln(µ/(1-µ)), as the link function. The response variable was a ratio (ranging between 0 and 1). We assessed the collinearity among the explanatory variables of each model by calculating the generalized variance inflation factor (GVIF) [36], using the R package car. We used the degrees of freedom (*df*) adjusted measure, GVIF^(1/(2 ∙ *df*))^, provided by the vif function in car. We found that this adjusted measure was less than two for all the sets of explanatory variables considered, which indicated that the explanatory variables were not highly correlated.

When fitting the models using the R function glmer, we included random effects to account for sources of spatial variability not captured by the fixed effects. We included county in which the nest observers were located and their unique NestWatch user identification numbers nested within county as random effects. These random effects accounted for random variability on a larger spatial scale and the variability in skill level among the individuals that reported data to NestWatch. Recognizing that these spatial random variability patterns varied by year, we nested the above random effects within year. This allowed the model to reflect the spatial variability that would not necessarily be the same from one year to the next. Using the Moran.I function from the R library ape, we calculated the global Moran’s I for each of the years 2007 through 2013 (we omitted 2006 due to the low number of observations). The analysis indicated that our model successfully accounted for spatial autocorrelation on the county-level of observer’s location, at level alpha = 0.05.

## Results

### Drought conditions

**Fig 1:**
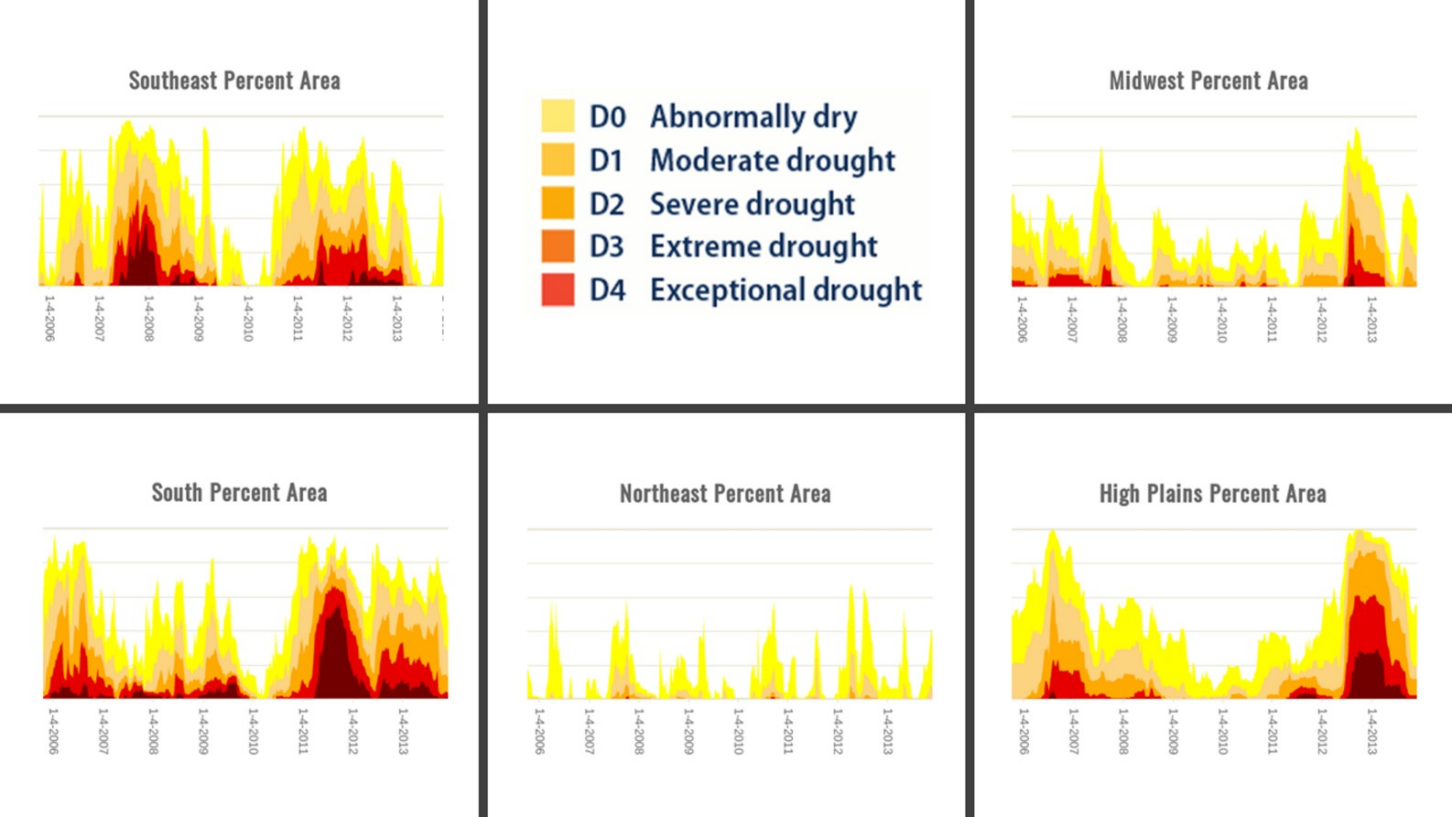
Percentage land coverage during drought conditions (2006–2013) by NADM climate regions encompassing the breeding range of Eastern Bluebirds (*Sialia sialis*). Modified from and courtesy of the National Drought Mitigation Center (NDMC). The U.S. Drought Monitor is jointly produced by the NDMC at the University of Nebraska-Lincoln, the U.S. Department of Agriculture, and the National Oceanic and Atmospheric Administration (https://droughtmonitor.unl.edu/Maps/MapArchive.aspx).

Abnormally dry (category D0) to drought conditions of varying severity (categories D1–D4) occurred within Eastern Bluebird nesting territories throughout the study period (Fig 1).

### Reproductive data

**Fig 2:**
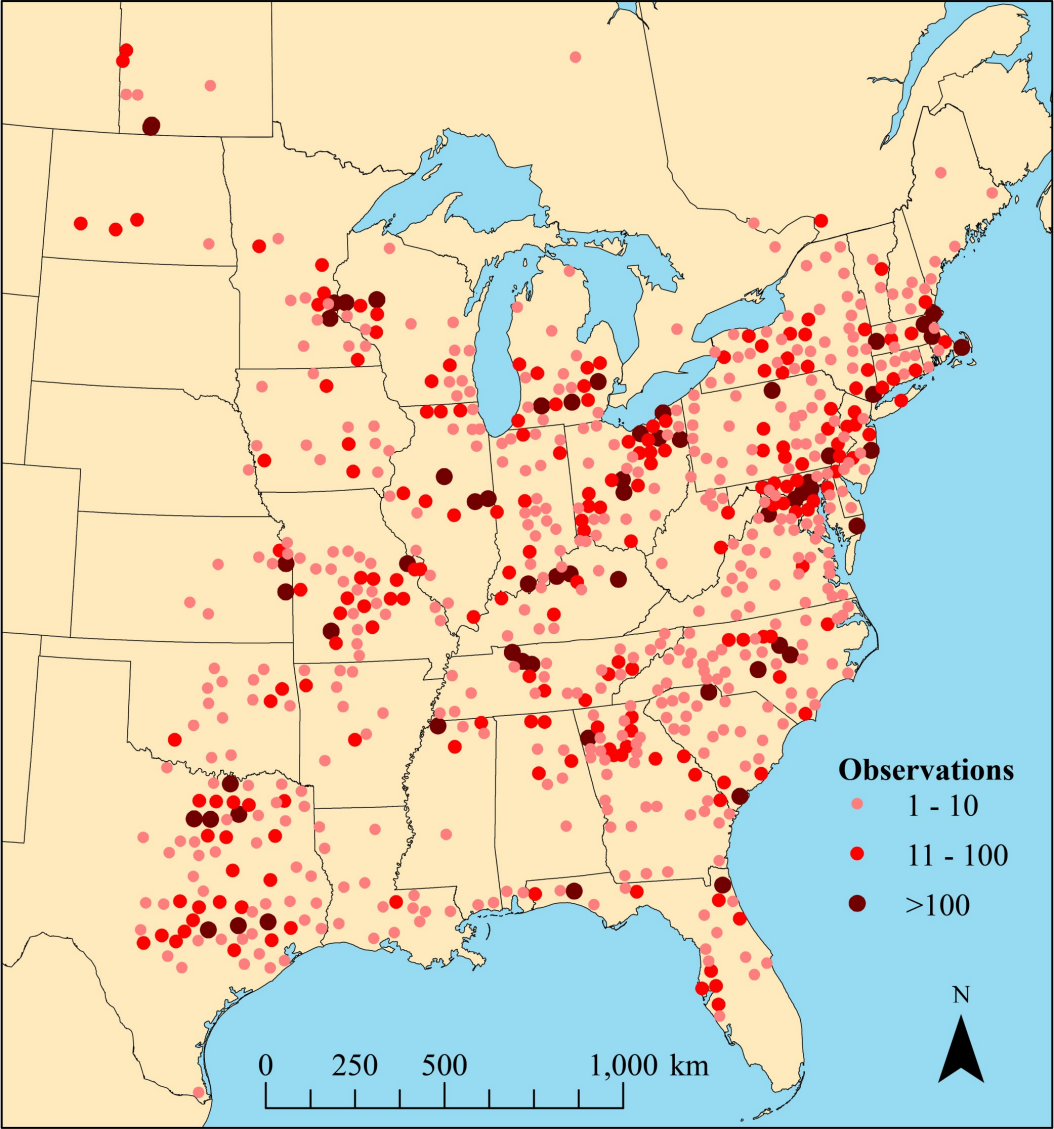
NestWatch observation sites within the North American breeding range of Eastern Bluebirds (*Sialia sialis*), 2006–2013. Created in ArcGIS 10.5.1 with mapping data obtained from the Canadian Open Government portal and U.S. Census Bureau public domain sources.

We received 24,368 individual nest observation records from NestWatch and included 708 observation records generated from our own nest box monitoring program. Following elimination of records with inaccuracies, 21,574 records were used for the analyses (Fig 2). The number of records was lowest in 2006 (*n* = 413), but increased yearly from *n =* 1876 in 2007 to *n =* 3,839 in 2012, before declining slightly in 2013 to *n* = 3,698.

### Clutch size

**Fig 3:**
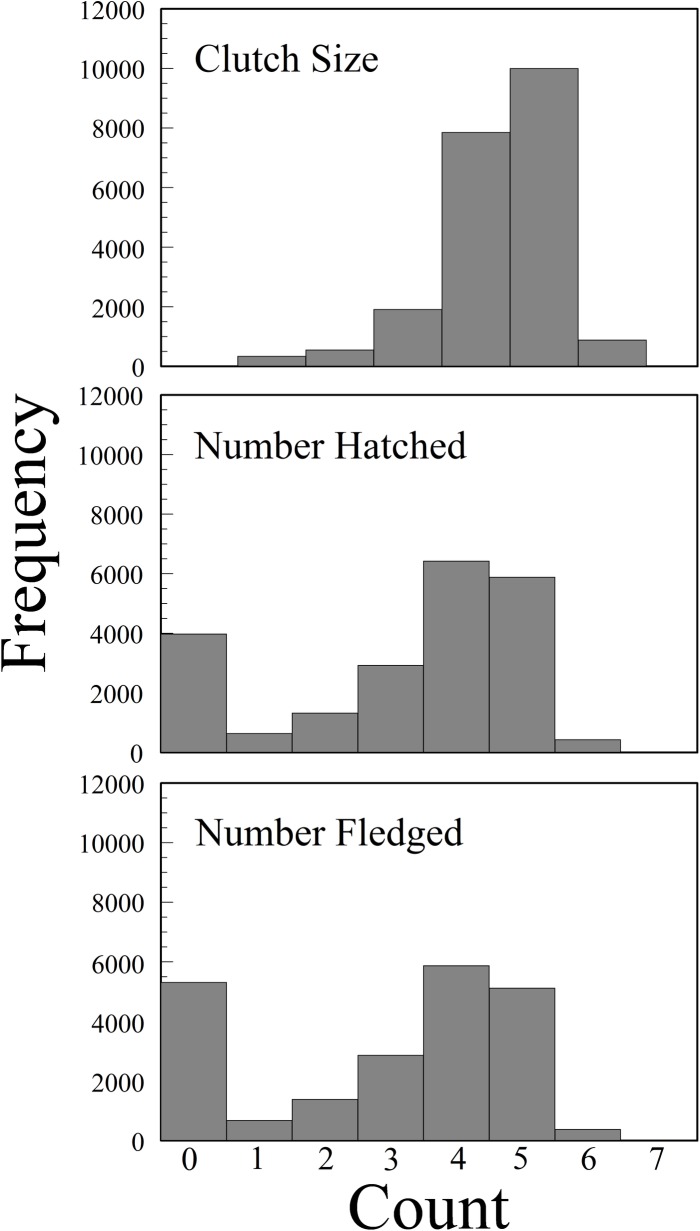
Frequency distributions of Eastern Bluebird clutch size, number hatched, and number fledged across their entire range in North America, 2006–2013.

Clutch size ranged from 1 to 7 eggs, with a mode of 5 and a mean of 4.37 (Fig 3). Clutch sizes peaked early in the breeding season and then declined as the season progressed (S1 Fig). Both latitude and the interaction between Julian lay date and latitude had significant effects on clutch size, though these were opposite in direction (i.e., latitude was positive and lay date x latitude was negative) (S1 Table). There was also a significant negative effect of longitude, but there was not a significant interaction between longitude and lay date (S1 Table and S1 Fig).

**Fig 4:**
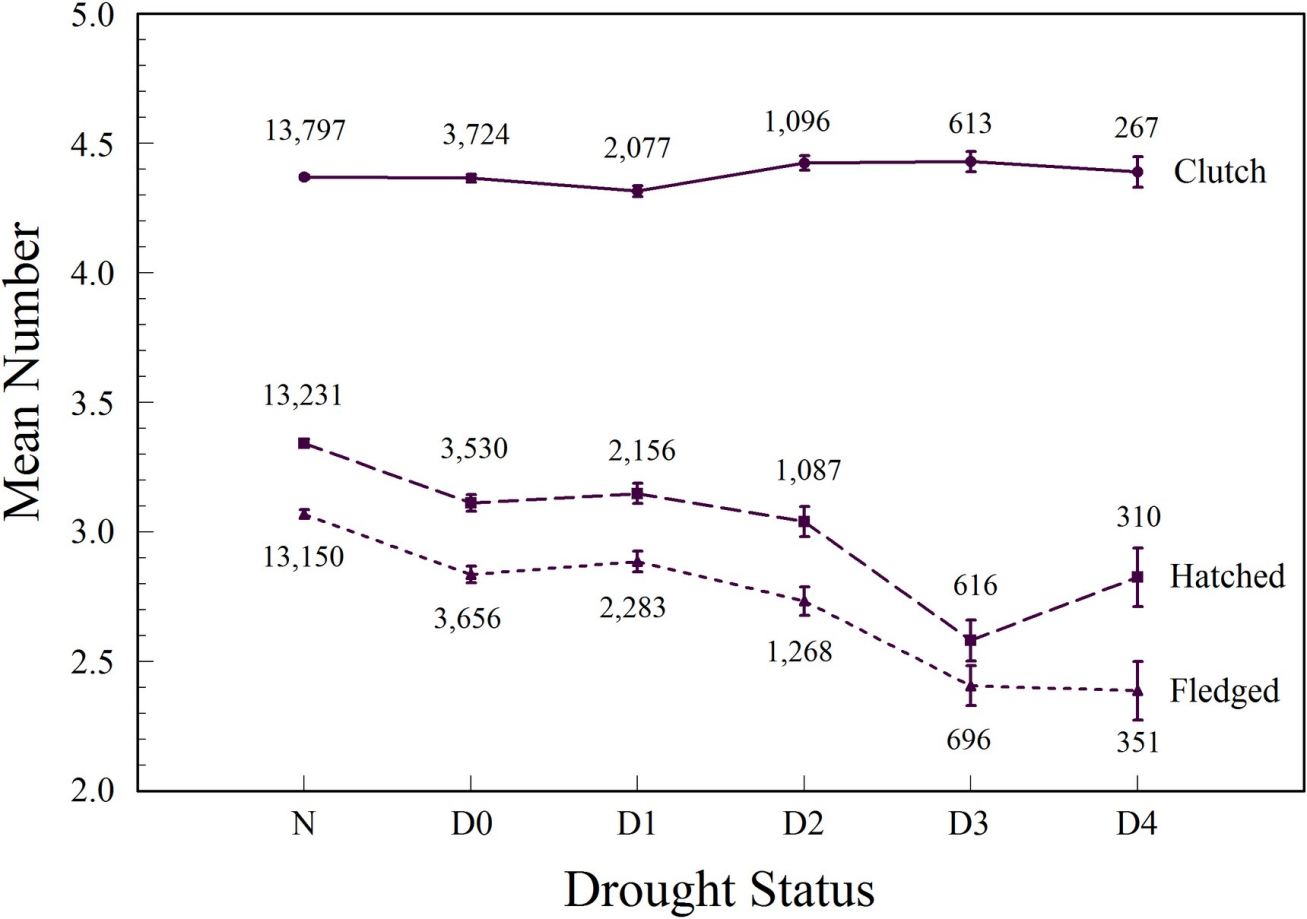
Mean number of Eastern Bluebird eggs laid (clutch size), eggs hatched, and young fledged by North American Drought Monitor drought status categories, 2006–2013.

The number of eggs per clutch did not decline with increasing drought severity (Fig 4). We report the coefficient estimates for the models including drought variables with clutch size as the outcome variable in Table 2. Three likelihood ratio (LR) tests were conducted, comparing each model to the baseline model that did not include drought status as an explanatory variable. The LR tests tested whether the effect of the drought status at the lay date, 30 days prior to lay date, and 60 days prior to the lay date, respectively, was significantly different from zero (H_0_: β = 0). The tests were executed by calling the R function anova, and the results showed no evidence in favor of rejecting the null hypotheses (*P* = 0.90, 0.65, and 0.86, respectively). We concluded that drought-status variables did not improve the fit of the model with clutch size as the outcome variable.

**Table 2:** Parameter *β* estimates (*b*) and probabilities (*P*) for three generalized linear models used to evaluate drought effects on Eastern Bluebird clutch size. Explanatory variables for all models are Julian lay date, latitude, longitude, standardized NDVI, and interactions between Julian lay date and latitude, and Julian lay date and longitude. Model 1 evaluates drought status during the Julian lay date and Models 2 and 3 evaluate drought status 30 days and 60 days prior to the Julian lay date, respectively.

Variable	Model 1		Model 2		Model 3	
	*b*	*P*	*b*	*P*	*b*	*P*
(Intercept)	1.474	0.000***	1.474	0.000***	1.473	0.000***
Lay Date	-0.079	0.000***	-0.08	0.000***	-0.08	0.000***
Latitude	0.011	0.005**	0.011	0.004**	0.011	0.004**
Longitude	-0.008	0.022*	-0.008	0.020*	-0.007	0.026*
Standardized *NDVI* for Lay Date	0.006	0.088	0.0079	0.068	0.0047	0.084
Drought Status D0	-0.003	0.733	0.006	0.51	-0.004	0.701
Drought Status D1	-0.005	0.4676	-0.002	0.867	0.003	0.776
Drought Status D2	0.015	0.319	0.013	0.421	0.012	0.437
Drought Status D3	0.006	0.756	-0.003	0.896	0.021	0.327
Drought Status D4	0.008	0.781	0.043	0.161	-0.007	0.827
Lay Date x Latitude	-0.016	0.000***	-0.016	0.000*	-0.016	0.000***
Lay Date x Longitude	0.0045	0.191	0.005	0.180	0.004	0.120

* *P* < 0.05;

** *P* < 0.01;

*** *P* < 0.001

Exponentiated regression coefficients (*e*^*b*^) can help with the interpretation of the coefficients in a particular model. Using the parameter estimates for the baseline model (S1 Table), the effect of lay date was significant, and the exponentiated coefficient (*e*^*b*^) for lay date, *e*^*b*^ = *e* ^-0.079^ = 0.924, gives the multiplicative effect of Julian date on clutch size when the date increases by one standard deviation. Whereas the multiplicative effect of latitude is increasing (*e* ^0.011^ = 1.011), the multiplicative effect of the interaction term is decreasing (*e* ^-0.016^ = 0.984). When interpreting the model results, it is important to keep in mind that all the numeric explanatory variables (Julian date, NDVI_std_, latitude and longitude) were standardized to have zero mean and a standard deviation of one prior to fitting the model.

### Hatching success rate

The modal number of hatching eggs was 4, followed by 5 and zero (Fig 3). The distribution was bimodal. The average number hatching was 3.23. Following a peak in March and April, the mean number of eggs hatching decreased as the season progressed. A seasonal decline in hatching was evident across latitudes; hatching peaked and declined earlier at lower latitudes compared to higher latitudes. This was supported in the baseline model by the significant interaction between hatch date and latitude (S1 Table and S1 Fig). For hatching rate, which is assumed to have a binomial distribution, the estimated coefficients in the various models represent the expected change in the log odds (logit) of hatching for a unit increase (one standard deviation) in the predictor variable, holding the other predictor variables constant. Exponentiation of the coefficient for latitude in the baseline model (*e* ^0.233^ = 1.263, for example) gives the odds ratio of an egg hatching versus an egg not hatching with one standard deviation increase in latitude. In contrast, odds of hatching (*e* ^-0.016^ = 0.984) decreased with increasing interaction. Longitude increased the odds of hatching (*e* ^0.105^ = 1.11), as did the interaction between longitude and hatch date (*e* ^0.151^ = 1.161). The effect of Julian hatch date alone was significant and negative (decreasing; *e* ^-0.225^ = 0.799) (S1 Table).

The number of hatched young declined from more than 3.3 to fewer than 3.0 hatching per nest with increasing drought status, especially for the two most extreme categories (Fig 4). As with clutch size, we also did nested, pairwise likelihood ratio tests comparing the models. Model 1 was a significant improvement over the baseline model (*Χ*^2^ = 45.342, *df* = 5, *P* << 0.0001). Likewise, Models 2 and 3 were significant improvements over the baseline model (*Χ*^2^ = 57.480, *df* = 5, *P* << 0.0001 and (*Χ*^2^ = 18.936, *df* = 5, *P* < 0.0020, respectively).

We report the model fitting results for hatch ratio for Models 1, 2, and 3 in Table 3. By exponentiation of the coefficients reported in the table, we calculated how the odds of eggs hatching (*e*^*b*^) were affected by each of the explanatory variables. The current date drought status results for Model 1 indicated that only the most severe levels of drought (D3 and D4), had a significant negative (decreasing) effect on the odds of hatching (relative to baseline level N; *e* ^-0.352^ = 0.703 and *e* ^-0.204^ = 0.816, respectively). Model 2 results, with the 30 days prior drought statuses added, were striking in that the effect (relative to baseline level N) of drought levels D0 through D4 were all significant and negative. In fact, the effect on the odds of hatching was more pronounced as conditions moved from D0 to D4 (the odds ratio is *e* ^-0.095^ = 0.909 for D0 and *e* ^-0.456^ = 0.634 for D4). For Model 3, only D0 was significant and negative (e ^-0.115^ = 0.892). Across all models, NDVI_std_ was positive and significant.

**Table 3:** Parameter *β* estimates (*b*) and probabilities (*P*) for three generalized linear models used to evaluate drought effects on Eastern Bluebird hatching success rate. Explanatory variables for all models include Julian lay date, latitude, longitude, standardized NDVI for the Julian hatch date, drought status during the Julian hatch date, and interactions between Julian hatch date and latitude and Julian hatch date and longitude. Models 2 and 3 evaluate drought status 30 days and 60 days prior to the Julian hatch date, respectively.

Variable	Model 1		Model 2		Model 3	
	*b*	*P*	*b*	*P*	*b*	*P*
(Intercept)	1.509	0.000***	1.571	0.000***	1.512	0.000***
Hatch Date	-0.219	0.000***	-0.219	0.000***	-0.224	0.000***
Latitude	0.227	0.000***	0.198	0.000***	0.241	0.000***
Longitude	0.087	0.007**	0.084	0.008**	0.094	0.003**
Standardized *NDVI* for Hatch Date	0.094	0.000***	0.096	0.000***	0.097	0.000***
Drought Status D0	0.044	0.154	-0.095	0.0016**	-0.115	0.000***
Drought Status D1	0.041	0.325	-0.117	0.007**	0.007	0.863
Drought Status D2	0.009	0.864	-0.356	0.000***	0.004	0.943
Drought Status D3	-0.352	0.000***	-0.46	0.000***	0.071	0.408
Drought Status D4	-0.204	0.002**	-0.456	0.000***	0.191	0.139
Hatch Date x Latitude	0.06	0.000**	0.058	0.000***	0.059	0.000***
Hatch Date x Longitude	0.15	0.000***	0.148	0.000***	0.152	0.000***

* *P* < 0.05;

** *P* < 0.01;

*** *P* < 0.001

### Fledging rate

The modal number of fledging young was 4, followed by zero and 5 (Fig 3). Again, the frequency distribution was bimodal, with more zero fledges than zero hatches. The average number fledged was 2.93. The interactions between fledging date and latitude and fledging date and longitude had a significant positive (increasing) effect on the odds ratios for fledging (i.e., *e* ^0.091^ = 1.096 and *e* ^0.148^ = 1.160, respectively) (S1 Table). The number fledging declined as the season progressed and from North to South and East to West (S1 Fig). In agreement with the hatching rate results, latitude and longitude were also positive and significant (*e* ^0.184^ = 1.202 and *e* ^0.06^ = 1.062, respectively), whereas Julian fledge date had a significant negative (decreasing) effect (*e* ^-0.118^ = 0.888) (S1 Table).

The number of fledged young declined from more than 3.0 fledges per nest to 2.4 fledges per nest with increasing drought status, especially for the two most extreme categories (Fig 4). As with clutch size and hatching success rate, we used models with current, 30-day, and 60-day prior drought statuses added to the baseline model, and summarize the results in Table 4. The results agreed closely with those for hatching success rate. First, the LR test results agree in that Model 1 (*Χ*^2^ = 55.907, *df* = 5, *P* << 0.0001), Model 2 (*Χ*^2^ = 56.983, *df* = 5, *P* << 0.0001), and Model 3 (*Χ*^2^ = 63.440, *df* = 5, *P* << 0.0001) were all significant improvements over the baseline model.

**Table 4:** Parameter *β* estimates (*b*) and probabilities (*P*) for three generalized linear models used to evaluate drought effects on Eastern Bluebird fledging success rate. Explanatory variables are Julian fledge date, latitude, longitude, standardized NDVI for the Julian fledge date, drought status during the Julian fledge date, and interactions between Julian fledge date and latitude and longitude, respectively. Models 2 and 3 evaluate drought status 30 and 60 days prior to the Julian fledge date.

Variable	Model 1		Model 2		Model 3	
	*b*	*P*	*b*	*P*	*b*	*P*
(Intercept)	1.026	0.000***	1.034	0.000***	1.072	0.000***
Fledge Date	-0.117	0.000***	-0.115	0.000***	-0.115	0.000***
Latitude	0.172	0.000***	0.164	0.000***	0.161	0.000***
Longitude	0.052	0.122	0.051	0.132	0.063	0.062
Standardized *NDVI* for Fledge Date	0.104	0.000***	0.102	0.000***	0.102	0.000***
Drought Status D0	0.028	0.35	0.053	0.065	-0.193	0.000***
Drought Status D1	0.102	0.01*	0.001	0.973	-0.003	0.95
Drought Status D2	-0.0875	0.097	-0.101	0.075	-0.117	0.034*
Drought Status D3	-0.322	0.000***	-0.40	0.000***	-0.339	0.000***
Drought Status D4	-0.313	0.000***	-0.463	0.000***	-0.363	0.002**
Fledge Date x Latitude	0.098	0.000***	0.097	0.000***	0.094	0.000***
Fledge Date x Longitude	0.139	0.000***	0.145	0.000***	0.149	0.000***

* *P* < 0.05;

** *P* < 0.01;

*** *P* < 0.001

Comparing the drought status results across models, we see that for Models 1 and 2, only the two most severe levels of drought (D3 and D4) had significant negative (decreasing) effects on the odds of fledging (relative to baseline level N) (the odds ratio was *e* ^-0.322^ = 0.725 for D3 in Model 1 and *e* ^-0.40^ = 0.671 for Model 2; for D4 the odds ratio was *e* ^-0.313^ = 0.731 for Model 1 and *e* ^-0.463^ = 0.629 for Model 2). For Model 1, D1 had a significant positive (increasing) effect (*e* ^0.102^ = 1.107).

For Model 3, with 60 days prior drought status added, levels D0, D2, D3, and D4 all had significant negative effects (relative to the baseline level N). Level D1 also had a slightly negative, but non-significant effect. The most severe drought levels (D3 and D4) had greater negative effects than those of the abnormally dry to severe drought levels (D0 and D2) (the odds ratio is *e* ^-0.193^ = 0.824 for D0 and *e* ^-0.339^ = 0.713 for D3). The effect of NDVI_std_ across all models for fledging rate was positive and significant.

## Discussion

We found that drought affected Eastern Bluebird reproduction in both expected and unexpected ways. Drought had no effect on clutch size, regardless of occurrence within 60 days prior to and during laying. Severity of drought had profound effects on both hatching and fledging success rates, with the most severe levels (D3 and D4) resulting in the greatest decreases in both variables. Drought occurring 30 and 60 days prior also had significant effects on hatching and fledging success; moreover, the most severe drought levels amplified decreases in hatching and fledging success rates.

### Clutch size

Results of our analyses were supportive of increased clutch sizes with increasing latitude, which is typical of most passerine birds [26, 27]. As suggested by Crick et al. [37] and supported by Dhondt et al. [28] and Cooper et al. [20], migratory, multi-brooded species breeding in more northern latitudes, including Eastern Bluebirds, produce larger clutches earlier in the breeding season compared to non-migratory populations breeding in southern latitudes. Southern populations also tend to produce larger clutches mid-season. Our results also followed this trend. Eastern Bluebird clutch size was not affected by any level of drought or correlated with standardized NDVI. Likewise, clutch sizes of Vesper Sparrows (*Pooecetes gramineus*) were not affected during historically severe drought conditions within the breeding range of that species [3]. The non-sensitivity of clutch size to drought may be due to its unpredictable and variable nature and evolutionary adaptations that favor production of an optimal clutch size even during environmentally challenging years [38]. Factors other than environment, such as female fitness, predation of the incubating female or eggs, or inaccuracies in the counting of eggs by observers, may have also influenced clutch-size data.

### Hatching success

Date of hatching significantly influenced hatch ratios such that hatch rate declined as the breeding season progressed. The observed pattern of increased hatching failure later in the breeding season, and at lower latitudes, is not uncommon among many bird species [20, 39]. Indeed, it is well-documented that hatching failure of Eastern Bluebird eggs follows this pattern [20, 28]. Our results suggest that this effect may be amplified during extreme and exceptional drought; in this study, considerably more nests (55%) experienced a 100% hatch rate when there was no drought (N) than when there were the most severe of droughts (levels D3 and D4) (less than 40% hatch rate) at the time hatching should have occurred. Abnormally dry to severe drought conditions (D0, D1, and D2) also appeared to influence hatch rate, but to a lesser degree. Drought 30 days prior to hatching had a more significant and negative effect, as evidenced by the greatly lowered odds of hatching under all drought statuses, while drought occurring 60 days prior had no or a reduced effect. This would be expected given that this time period would be well before clutch initiation.

The decreased hatching success we documented with increasing drought levels occurring 30 days prior to hatching may have resulted from factors associated with higher drought-associated temperatures during the pre-incubation and incubation period. We did not, however, include ambient temperature in our analyses. It is possible, although not necessarily so, that there were higher than normal temperatures occurring concurrently with drought conditions. For example, Karnieli et al. [40] demonstrated a negative relationship between NDVI and land surface temperature (LST) during the May to October period, which partially coincides with the latter portion of the bluebird breeding season. Changes in female incubation behavior, loss of egg viability, embryo mortality, or a combination are associated with higher ambient temperatures occurring before and during incubation [29, 41, 42]. It has also been suggested that higher temperatures reduce egg viability via changes in embryo development, but not necessarily embryo mortality [29]. The egg-viability hypothesis suggests that egg viability varies with latitude, with clutch initiation date, and with the sequence at which eggs are laid [43]. In some species, prolonged exposure to high ambient temperature, as would occur with the first eggs laid in a clutch, decreased egg viability and reduced hatching success [42, 43, 44].

### Fledging success

Fledging success is influenced by many factors. Asynchronous hatching within a clutch, for example, results in a higher probability that the youngest nestling will die of starvation [45, 46]. Cooper et al. [20, 29] found that female Eastern Bluebirds breeding at lower latitudes tended to initiate incubation before clutch completion, which would increase the likelihood of asynchronous hatching and loss of the youngest nestlings. Our observation of decreased fledging success with increasing drought conditions prior to and at the time of fledging, especially when drought occurred up to 60 days prior to fledging, was possibly due to associated decreases in available food supply for both the nestlings and their parents. Smith [47] noted changes in insect densities and species composition along with a decline in numbers of insectivorous birds during drought. It is well established that the reproduction and abundance of herbivorous insects is highly sensitive to the availability and quality of plant foliage [48]. Although the effects of precipitation on insect reproduction and survival have not been extensively studied [49], Thacker et al. [50] found variable rainfall during a critical stage of aphid reproduction to have a negative effect on both foliage quality and aphid abundance. It is thus likely that the timing and severity of drought negatively impacted insect populations that are critical for nestling survival.

### The power of citizen science data

Reproductive data acquired over a 7-year period through NestWatch and from our own study site, and NDVI and NADM data provided the opportunity to examine drought effects across the Eastern Bluebird North American breeding range. While smaller studies conducted solely by experienced researchers yield more accurate data and have value for exploring localized effects, the field time required for a broader study is prohibitive. For example, reproductive data collected from our study site expended over 840 field hours on average for each approximately 173-day field season. Data entry was also time intensive. The more than 24,000 NestWatch records we received were easily imported into a database and then scrubbed of questionable records using simple SQL queries. Consequently, our data set was robust and efficient in terms of sample size and time expenditure, respectively.

## Conclusion

Our results demonstrated distinct drought impacts on certain aspects of Eastern Bluebird reproduction. We found a clear association between occurrence of extreme or exceptional droughts during critical periods of the Eastern Bluebird nesting cycle and decreases in both hatching and fledging success. Eastern Bluebirds are a species of least concern [30] and thus not likely to experience widespread changes in population densities due to drought alone. We suggest, however, that drought effects could be significantly more deleterious for species in decline. Studies based on citizen science-generated data for other species combined with standardized climate date could provide additional support for this observation.

## Supporting information

S1 Fig Three-dimensional plots of Eastern Bluebird clutch size, number hatched, and number fledged by Julian date and latitude (A) and Julian date and longitude (B).
(PDF)

S1 Table Parameter *β* estimates (*b*) and probabilities (*P*) for the baseline model used to evaluate effects of Julian date, latitude, longitude, NDVI_std_, and interactions between date and latitude and date and longitude on Eastern Bluebird clutch size, hatch rate, and fledge rate. (PDF)
