# Supplementary material for: Reproductive success of Eastern Bluebirds (Sialia sialis) varies with the timing and severity of drought
Source: PLoS One. 2019 Aug 9;14(8):e0214266. doi: 10.1371/journal.pone.0214266 (PMC6688811; doi:10.1371/journal.pone.0214266)

S1 Fig. 3D plots of Eastern Bluebird clutch size, number hatched, and number fledged by Julian date and latitude (A) and Julian date and longitude (B).

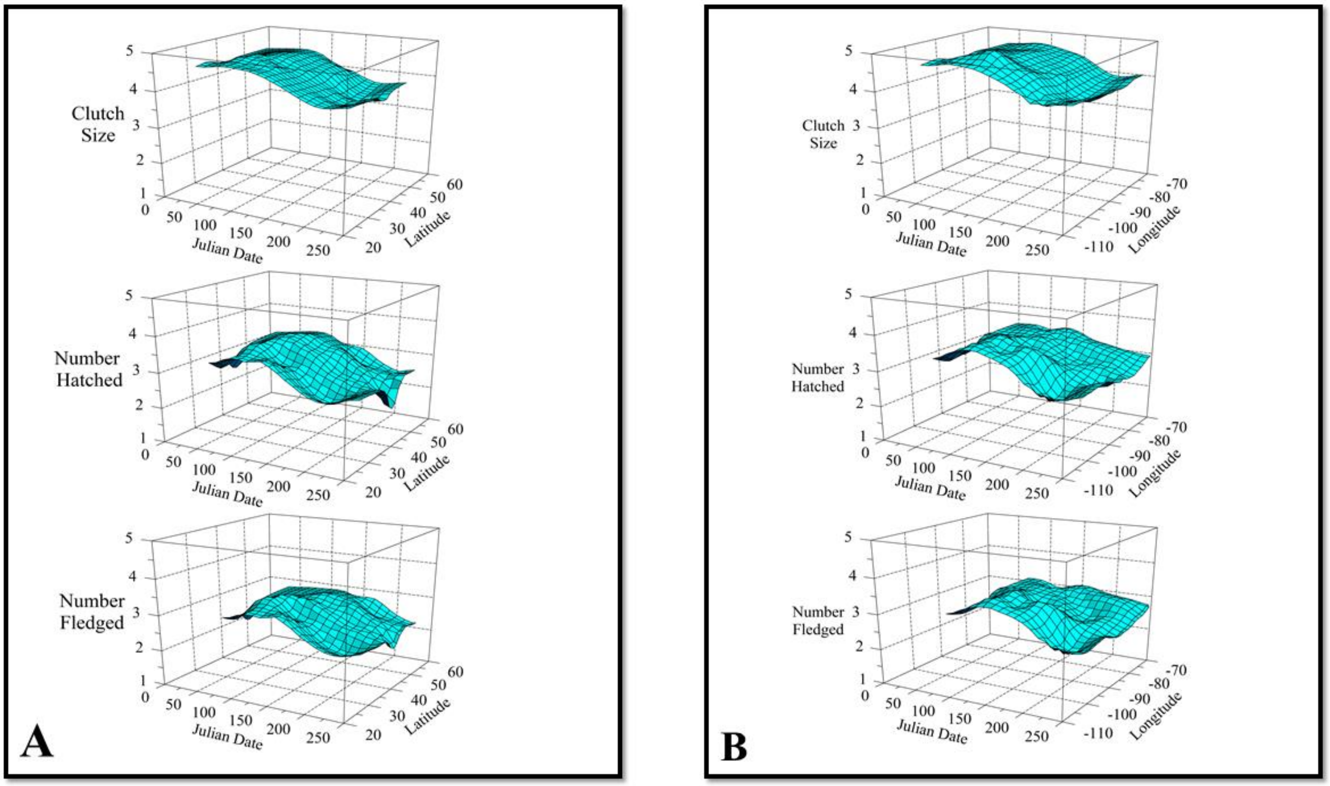

Supplement: S1 Fig — Three-dimensional plots of Eastern Bluebird clutch size, number hatched, and number fledged by Julian date and latitude (A) and Julian date and longitude (B). (PDF) [file pone.0214266.s001.pdf]
