# Supplementary material for: Reproductive success of Eastern Bluebirds (Sialia sialis) varies with the timing and severity of drought
Source: PLoS One. 2019 Aug 9;14(8):e0214266. doi: 10.1371/journal.pone.0214266 (PMC6688811; doi:10.1371/journal.pone.0214266)
Supplement: S1 Table — (PDF) [file pone.0214266.s002.pdf]

S1 Table. Parameter *β* estimates (*b*) and probabilities (*P*) for the baseline model used to evaluate effects of Julian date, latitude, longitude, $NDVI_{std}$, and interactions between date and latitude and date and longitude on Eastern Bluebird clutch size, hatch rate, and fledge rate.

	Clutch Size		Hatch Rate		Fledge Rate	
Variable	Estimate	Pr(>|z|)	Estimate	Pr(>|z|)	Estimate	Pr(>|z|)
(Intercept)	1.74	0.00***	1.53	0.00***	1.023	0.00***
Julian date	-0.076	0.00***	-0.225	0.00***	-0.118	0.00***
Latitude	0.011	0.005**	0.233	0.00***	0.184	0.00***
Longitude	-0.008	0.016*	0.105	0.001**	0.06	0.07
$NDVI_{std}$	0.007	0.09	0.088	0.00***	0.107	0.00***
Date x latitude	-0.016	0.00***	0.056	0.00***	0.09	0.00***
Date x longitude	0.004	0.199	0.15	0.00***	0.148	0.00***

significance *** <0.000; ** <0.001; *<0.05
